# Host and Viral Genetic Correlates of Clinical Definitions of HIV-1 Disease Progression

**DOI:** 10.1371/journal.pone.0011079

**Published:** 2010-06-11

**Authors:** Concepción Casado, Sara Colombo, Andri Rauch, Raquel Martínez, Huldrych F. Günthard, Soledad Garcia, Carmen Rodríguez, Jorge del Romero, Amalio Telenti, Cecilio López-Galíndez

**Affiliations:** 1 Centro Nacional de Microbiología, Instituto de Salud Carlos III, Madrid, Spain; 2 Institute of Microbiology, University Hospital Center, University of Lausanne, Lausanne, Switzerland; 3 University Clinic of Infectious Diseases, University Hospital Bern and University of Bern, Bern, Switzerland; 4 Division of Infectious Diseases and Hospital Epidemiology, University of Zurich, Zurich, Switzerland; 5 Centro Sanitario Sandoval, IMSALUD Comunidad Autónoma de Madrid, Madrid, Spain; Institut Pasteur, France

## Abstract

**Background:**

Various patterns of HIV-1 disease progression are described in clinical practice and in research. There is a need to assess the specificity of commonly used definitions of long term non-progressor (LTNP) elite controllers (LTNP-EC), viremic controllers (LTNP-VC), and viremic non controllers (LTNP-NC), as well as of chronic progressors (P) and rapid progressors (RP).

**Methodology and Principal Findings:**

We re-evaluated the HIV-1 clinical definitions, summarized in Table 1, using the information provided by a selected number of host genetic markers and viral factors. There is a continuous decrease of protective factors and an accumulation of risk factors from LTNP-EC to RP. Statistical differences in frequency of protective *HLA-B* alleles (p-0.01), *HLA-C* rs9264942 (p-0.06), and protective *CCR5/CCR2* haplotypes (p-0.02) across groups, and the presence of viruses with an ancestral genotype in the “viral dating” (i.e., nucleotide sequences with low viral divergence from the most recent common ancestor) support the differences among principal clinical groups of HIV-1 infected individuals.

**Conclusions:**

A combination of host genetic and viral factors supports current clinical definitions that discriminate among patterns of HIV-1 progression. The study also emphasizes the need to apply a standardized and accepted set of clinical definitions for the purpose of disease stratification and research.

## Introduction

A continuous spectrum of disease progression rates characterizes HIV-1 infection. This observation led to various definitions of clinical progression that are widely used for the designation of patient subsets: from elite controllers to rapid progressors. It is not well established how clinical definitions relate to various determinants of pathogenesis. In particular, a number of host genetic, immune and virological factors have been associated with various patterns of disease progression [1], [2].

The contribution of host factors to viral load and to disease progression has now been established at genome level [3], [4]. Genetic variants, validated in several genome-wide association studies, explain 13% of the observed variability in HIV-1 viremia [3]. The addition of gender, age and residual population structure to the genetic model increases the figure up to 22% [4]. Estimates could be improved with more complex and diverse predictive models.

Among viral factors studied for their association with HIV-1 disease progression, RNA viral load is the best marker [5]. In addition, HIV DNA levels in peripheral blood mononuclear cells (PBMCs) have prognostic value early in infection [6]. Viral phenotypes related to clinical progression include the CXCR4 or CCR5 coreceptor usage; with CXCR4 use associated with more rapid progression to AIDS [7]. Several studies describe a correlation between disease progression and the extent of HIV-1 genetic variation [8], [9], [10]. From an evolutionary perspective, the analysis of multiple isolates from the Spanish HIV-1 epidemic permitted the inference of the “viral dating” of isolate sequences as an estimation of viral evolution [11]. A subset of long term non-progressors (LTNPs) carries ancestral viruses (i.e. the estimated date of the viral nucleotide sequences is close to the seroconversion time), because of the control of viral replication. In contrast, individuals with continuous viral evolution carry viruses with modern dating (i.e. close to the sampling time), reflecting the ongoing process of viral divergence [12].

Using this background knowledge, the study aims at re-evaluating broadly applied clinical definitions of disease progression: LTNP elite controllers (LTNP-EC), viremic controllers (LTNP-VC), viremic non controllers (LTNP-NC), chronic progressors (P) and rapid progressors (RP) under the information provided by a selected number of viral and host genetic characteristics. Importantly, the study is not a de-novo genetic or viral study, rather the combined application of well established knowledge that should result in a clear readout in small sets of individuals. The analysis emphasizes, in particular, the characteristics of the least investigated group of individuals, the rapid progressors.

## Materials and Methods

### Ethics statement

The study was approved by the Comite de Etica del Centro Sanitario Sandoval, C/Sandoval 7, Madrid 28010 and by the University of Lausanne, Faculty of Medicine Commission d' Ethique de la Recherche Clinique rue du Bugnon 21, 1005 Lausanne.

### Study subjects

We included 64 treatment naïve individuals, from the Centro Sanitario Sandoval (IMSALUD, Madrid, Spain), and from the Swiss HIV Cohort Study (www.shcs.ch) that fulfilled criteria for the various clinical progression definitions (Table 1). In the absence of standardized definition of the various clinical progression profiles, we based strict definitions on published literature [1], [2], [13], [14], and on their broad clinical use. LTNPs patients, including elite and viremic controllers, from Spain met the strictest definition of more than 10 years of infection and undetectable or low viremia. Participants gave informed genetic consent for the study (which was oral and general for different type of studies for participants with a long term follow up in the Centro Sanitario Sandoval), and the study and the consents was approved by the Committees of the two Centers. Seroconversion date was estimated from either a documented negative test, less than two years before the first documented positive test, or biological criteria of primary infection: incomplete western blot and/or positive p24 Ag and/or high viremia (>1 million copies per milliliter of blood). Seroconversion date was defined as the mid-point between the two dates. These individuals have not been included in previous genetic studies, with the exception of 10 chronic progressors [3].

**Table 1:** Definitions of the five clinical progression groups.

**LTNP-EC**	•Asymptomatic HIV Infection over 10 year after seroconversion•Plasma HIV RNA levels without ART that are below the level of detection for the respective assay (e.g., <75 copies/mL by bDNA or <50 by ultrasensitive PCR).•Isolated episodes of viremia up to 1000 copies/mL as long as they are not consecutive and represent the minority of all available determinations.•Longitudinal HIV RNA that includes a minimum of 3 determinations, in the absence of antiretroviral agents, which span at least a 12-month period.
**LTNP-VC**	•Asymptomatic HIV Infection over 10 year after seroconversion.•Plasma HIV RNA levels without ART that are equal or below 2000 copies/mL.•Isolated episodes of viremia above 2000 copies/mL as long as such episodes represent the minority of all available determinations.•Longitudinal HIV RNA that includes a minimum of 3 determinations, in the absence of ART, which span at least a 12-month period.
**LTNP-NC**	•Asymptomatic HIV Infection over 10 year after seroconversion•Plasma HIV RNA levels above 2.000 copies/mL without ART, in more than 50% of the samples.
**P**	•Symptomatic infection or initiation of ART within 10 years after seroconversion•Longitudinal HIV RNA that includes a minimum of 3 determinations, in the absence of ART, with a viral set point above 2000 copies/mL
**RP**	•≥2 CD4 T cell measurements below 350/mm^3^ within 3 years after seroconversion, with no value ≥350 afterwards in the absence of ART.•And/or, ART initiated within 3 years after seroconversion, and at least one preceding CD4 < 350/mm^3^.•And/or, AIDS or AIDS-related Death within 3 years after seroconversion and at least one preceding CD4<350/mm^3^.

LTNP-EC: long term non-progressor, elite controllers; LTNP-VC: long term non-progressor, viremic controllers; LTNP-NC: long term non-progressor, viremic non controllers; P: chronic progressors, RP: rapid progressors, ART: antiretroviral therapy. Clinical groups summarize different definitions from the literature [1], [2], [13], [14].

### Host genetic characterization

Host genetic variants were chosen on the basis of genome-wide association studies [3], [4], or selected from the literature according to the quality of their supporting evidence (www.hiv-pharmacogenomics.org). These included *HLA-B* alleles associated with protection or progressive disease, the *HCP5* rs2395029 allele in linkage disequilibrium with HLA-B*5701, the *HLA-C-35* (rs9264942) variant, *ZNRD1* rs9261174 and *HLA-A10 serogroup* alleles in linkage disequilibrium, *CCR5 Δ32* (rs333), *CCR2 V64I* (rs1799864), *CCR5* haplotypes, and copy number variation of *CCL3L1*. *CCR5* haplotypes were constructed according to the published nomenclature [15]; considering 8 polymorphisms in the *CCR5/CCR2* promoter and coding region (rs2856758, rs2734648, rs1799987, rs1799988, rs1800023, rs1800024, rs333, rs1799864); *CCR5_P1* haplotype is carried by *HHE*, HHG*1 and HHF*1 haplotypes. *HLA* typing was done by sequencing, and SNP (single nucleotide polymorphism) analysis was done by TaqMan. Additive unweighted genetic scores were used to compile genetic information [16]. In this model the impact of each allele is assumed to be the same except for the sign (i.e. alleles with a protective effect were added, and risk alleles were subtracted. A simplified score was applied: *CCR5 Δ32* [score 0,1], *CCR2 V64I* [0,1,2], *CCR5 P1* homozygous [0,−2], *HLA-C–35* [0,1,2], and protective *HLA-B+* [0,1,2] and detrimental *HLA-B-* alleles [0,−1,−2]). This simplified procedure was also applied to estimate allelic frequencies for groups (e.g. protective *HLA B* alleles).

### Viral load and DNA quantification, PCR amplification and nucleotide sequencing

Plasma HIV-1 RNA viral load (VL) was quantified with the Branched DNA Siemens versant HIV RNA 3.0 assay (bDNA), or the Roche Amplicor with a detection limit of 75 and 50 copies/ml respectively. Viral set point was defined as the average of viral load results after assessment of each individual data and elimination of VL outliers after seroconversion and before antiretroviral therapy [3]. PBMC-associated DNA was obtained from 10^7^ cells by standard methods. HIV DNA was amplified in the C2-V5 region of the *env* gene as described [11], [12] and sequenced in both directions. Quantification of DNA, estimated by limiting dilution PCRs, was expressed as number of copies per million peripheral mononuclear cells (PBMC).

### Phylogenetic analysis of the *env* gene

For viral phylogenetic analysis and X4/R5 genotype, a 210 bp fragment in *env* gene from the distal position of C2 to the middle of C3 was obtained for each participant. Bulk nucleotide sequences were edited using the SeqMan version 3.61 (Inc. Dnastar, Madison, Wis). An estimation of the “viral dating” of nucleotide sequences was established, as previously reported by Bello et *al* [11], [12], according to the genetic distance to the reconstructed origin of the HIV-1 subtype B Spanish epidemic and assuming a relaxed molecular clock. The estimated dating time of the ancestral samples studied differs from the sampling time up to 15 years. A similar dating time was obtained when considering as most recent common ancestor (MRCA), the ancestral virus of the subtype B epidemic in the Los Alamos Database (USA).

The CXCR4 or the CCR5 phenotype was inferred from the V3 amino acid bulk sequences by the PSSM algorithm in Jensen *et al* [17] in (http://indra.mullins.microbiol.washington.edu/pssm/) site.

## Results

The study individuals represent the five clinical definitions summarized in Table 1: LTNP-EC (n = 9), LTNP-VC (n = 7), LTNP-NC (n = 14), P (n = 10) and RP (n = 24). The characteristics of the study participants are presented in Table S1.

**Figure 1:**
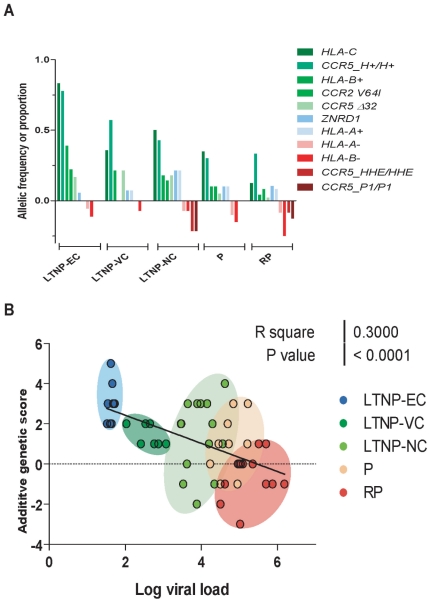
Distribution of protective and risk alleles and genetic score across clinical definitions of disease progression. **Panel A**. Participants fulfilled criteria for the definition of long term non-progressor elite controllers (LTNP-EC), LTNP viremic controllers (LTNP-VC), LTNP viremic non controllers (LTNP-NC), chronic progressors (P), and rapid progressors (RP). Analysis included genotyping for *HLA-C-35* (rs9264942) (HLA-C), *CCR5 Δ32* (rs333) and *CCR2 V64I* (rs1799864) and other polymorphisms in the *CCR5* promoter region (rs2856758, rs2734648, rs1799987, rs1799988, rs1800023, rs1800024) that define protective (*CCR5_H+*) or risk haplotypes (*CCR5_HHE or CCR5_P1*), *ZNRD1* rs9261174, and alleles in the *HLA-A* and *HLA-B* loci, including protective (*HLA−A+, HLA−B+*) and risk (*HLA−A−, HLA−B−*) alleles. For *CCR5* haplotypes, the proportion of individuals carrying protective or risk genotypes are reported. The allelic frequency is represented for other genetic markers. For clarity, protective factors are represented on the positive Y-axis and risk factors on the negative Y-axis. The specific alleles and haplotypes considered are indicated in the text and Table S1. **Panel B**. Correlation between viral load and a simple additive genetic score that includes the most valuable genetic markers as explained in Materials and Methods section: The distribution of the various clinical definition groups in color coded (LTNP-EC, blue; LTNP-VC, dark green; LTNP-NC, light green; P, orange; RP, red).

**Figure 2:**
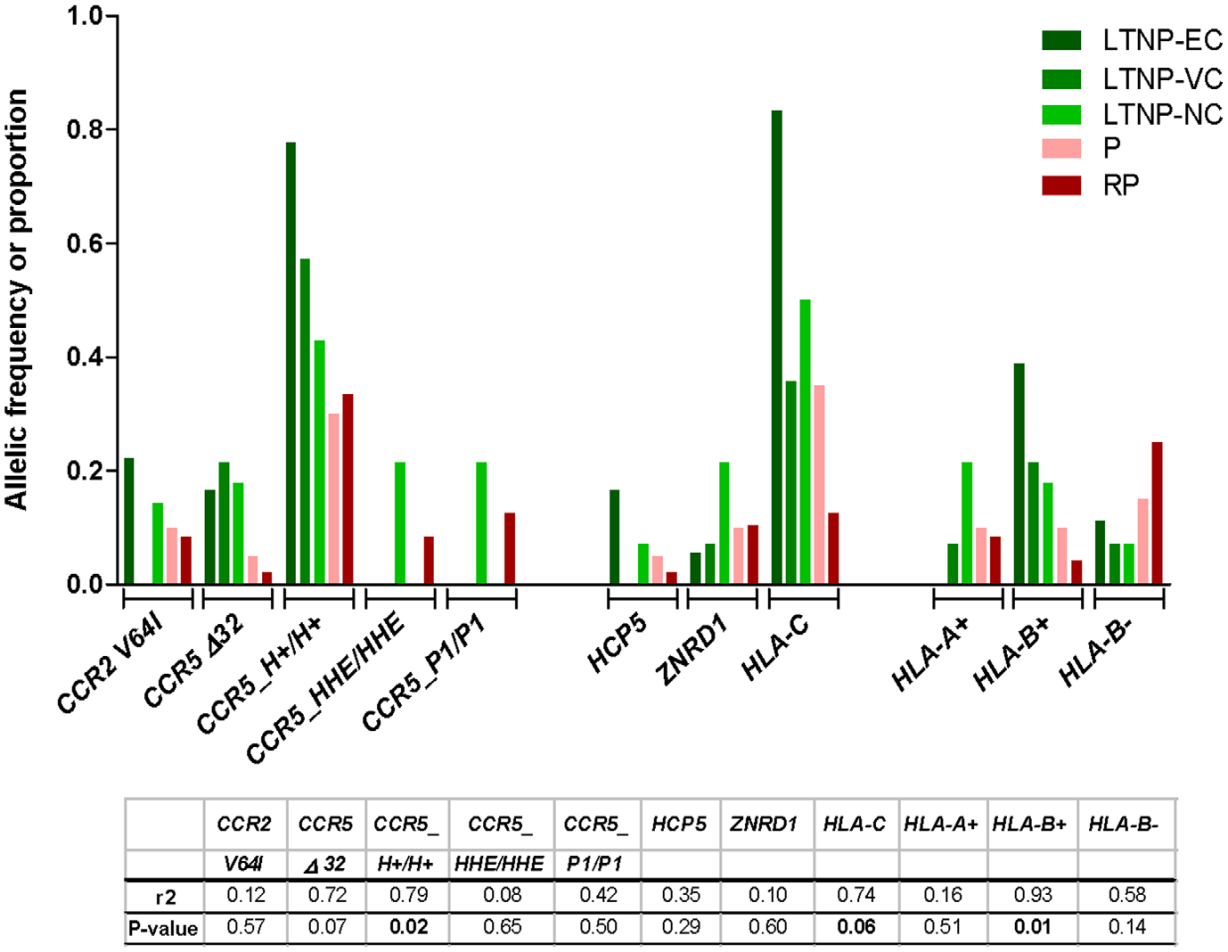
Statistical analyses of the distribution of protective and risk alleles across clinical definitions of disease progression. Patients who fulfilled criteria for the definition of long term non-progressor elite controllers (LTNP-EC), LTNP viremic controllers (LTNP-VC), LTNP non controllers (LTNP-NC), chronic progressors (P), and rapid progressors (RP). Analysis included genotyping for *HLA-C-35* (rs9264942) (HLA-C), *CCR5 Δ32* (rs333) and *CCR2 V64I* (rs1799864), *CCR5* haplotypes (inferred from *CCR5 Δ32*, *CCR2 V64I and* rs2856758, rs2734648, rs1799987, rs1799988, rs1800023, rs1800024) which define protective (*CCR5_H+/H+*) or risk haplotypes (*CCR5_HHE/HHE or CCR5_P1/P1*), *ZNRD1* rs9261174, *HLA* alleles in the *HLA-A* and *HLA−B loci*, including protective (*HLA−A+, HLA−B+*) and risk (*HLA−A−, HLA−B−*) alleles, as well as *HCP5* rs2395029 allele in linkage disequilibrium with HLA−B*5701. The allele frequency of each analyzed marker is presented, with the exception of *CCR5* haplotypes, where the proportion of individuals is reported. The specific alleles and haplotypes are shown in Table S1.

From LTNP-EC to RP, clinical definitions were associated with changes in the frequency (depletion) of protective host factors, in particular the *CCR5* protective haplotypes (*CCR5_H*+/H+, proportion decreasing from *f* = 0.78 in EC to 0.33 in RP), the *HLA-B* protective alleles (B*2705, *5701, *5101, *1302, codified as *HLA-B*+, decreasing from *f* = 0.39 to 0.04), and the *HLA-C-35* rs9264942 variant (decreasing from *f* = 0.83 to 0.13). The inverse situation occurred with host markers related with rapid progression, in particular *CCR5_P1* homozygosity (proportion increasing from 0 in EC to 0.13 in RP), and *HLA-B* risk alleles (B*1801, HLA-B*35Px alleles and *B22* serogroup, increasing from *f* = 0.11 to 0.25), Figure 1A. In regression analysis, several of the markers displayed significant statistical association in their frequency distribution across clinical definitions: protective *HLA-B*+ (r2 = 0.93, p = 0.01) and *CCR5_H+/H+* (r2 = 0.79, p = 0.02), and trend association for *HLA-C-35* (r2 = 0.75, p = 0.06) and *CCR5 Δ32* (r2 = 0.72, p = 0.07); Figure 2. Consistent with recent data [18], copy number of *CCL3L1* did not discriminate among clinical groups (average copies per diploid genome; LTNP-EC, 2.1; LTNP-VC, 2.6; LTNP-NC, 2.1; P, 1.6; RP, 2.2).

Selected viral factors previously associated with HIV clinical progression were included in our analysis: viral load, viral DNA load, viral dating, and X4/R5 genotype (Table S1). As expected, there was a progressive increase in the viral load from LTNP-EC to the RP. The increase in viral load was significantly associated (p value<0.0001) with a simple additive score that included the most valuable genetic markers (*HLA-C -35, CCR5 Δ32, CCR2 V64I, CCR5_P1/P1*, and *HLA-B+* and *B-* alleles), Figure 1B. The average additive genetic score was 3.0 for EC, 1.5 for LTNP viremic controllers, 1.3 for LTNP-NC, 0.9 for P, and −0.3 for RP (p<0.0001). An increase in proviral DNA values was also associated with the various clinical definitions. Proviral load was extremely low in LTNP-EC and LTNP-VC (in general, <5 copies/10^6^ PBMCs). LTNP-NC were, in general, above 30 copies/10^6^, and RP showed values 100 times higher values than the LTNP-EC and LTNP-VC (Table S1). Thus, although a precise discrimination across groups is limited by evidence of overlap of close disease strata, the emphasis is placed on the dosing of multiple protective and risk alleles that define progression.

An important difference between groups was the presence or absence of evolution in the viral quasispecies. Chronic and rapid progressors, presenting a continuous high viral replication carried, as expected, modern viruses. In contrast, among LTNP-EC and LTNP-VC, individuals maintained ancestral virus genotypes, close to the transmitted virus [11], [19], [20] although residual viral replication may occur [21], [22], [23]. The switch from the ancestral to the modern genotype occurred within the group of LTNP-VC (Table S1); differences in allelic frequency of various host factors distinguished these two groups. The additive genetic score was 2.6 among individuals carrying ancestral viruses, vs 1.5 for LTNP carrying modern viruses, p = 0.08 (Figure 1B).

The possible contribution of the X4/R5 genotype to explain the differences observed between groups was also assessed. Receptor use, deduced from the V3 amino acid sequence, was statistically consistent with an R5 phenotype in viruses from all the studied patients (Table S1).

## Discussion

There are different classifications of HIV-1 patients because of the distinct criteria used [13]. Classifications based in clinical data differentiate LTNPs, P and RP [1]. Consideration and inclusion of viral load measurements allows the definition of additional sub-categories: LTNP elite controllers, LTNP viremic controllers and LTNP viremic non-controllers [2].

This study shows that these broadly used clinical definitions of HIV-1 disease progression are generally supported by the pattern of distribution and enrichment of viral and host genetic factors. Among them, the frequencies of protective *HLA-B* alleles, *HLA-C-35* rs9264942, and protective *CCR5/CCR2* alleles as well as the ancestral/modern genotype were the factors that best discriminated among groups. The least distinct groups are the LTNP with (LTNP-VC) or without (LTNP-NC) effective control of viremia that cannot be readily separated on the basis of host genetic markers. Analysis of the characteristics of viral sequences permitted, however, to distinguish within LTNP-VC a subset of individuals with viruses with ancestral dating – a characteristic of the LTNP-EC. The lack of viral evolution, which is the basis of the ancestral characteristic of the sequences, reflects the strict control of viral replication. The ancestral genotype in LTNP-VC could have prognostic value; but the limited number of individuals in this group, that differ solely on the basis of viral dating, does not allow for greater precision on whether the split within this category will be of clinical consequence.

The study emphasizes the characteristics of the group described as RP: very high viral load, few protective host factors and an increased presence of host genetic progression factors. Depending on the definition used, approximately 10% of HIV-infected individuals progress to AIDS within the first two or three years of HIV-1 infection. Recently, Dalmau *et al* described detailed genetic, virologic and clinical analyses of two who progressed in less than one year [24]. A combination of immunological, genetic, and viral factors were found contributing to the extremely pathogenic infection, including the detection of highly replicative dual tropic X4/R5 viruses [24]. None of the rapid progressors in the present study had viruses with sequence features associated with the presence of the more pathogenic X4 variant [7].

The study assessed also the host genetic determinants that associate with complete control of viral replication, as defined by the identification of ancestral viral populations –viruses that did not or minimally evolve from the founder ancestral sequence. Individuals carrying ancestral viruses have an enrichment of host protective viruses compared with those carrying modern viral.

Viral dating methodology is a rapid approach to measure, within a given individual and in a single sample, viral evolution. This methodology has limitations [25] but it has shown it usefulness for the classification of LTNP [11], [12]. Although there is a global control of viral replication which maintains the characteristic ancestral genotype, several reports have provided evidence of residual viral replication in elite controllers [21], [22], [26]. The contribution of this residual viremia to viral evolution in ancestral patients was recently estimated; it showed that the percentage of evolved sequences represent, in general, less than 2% of the sequences in the quasispecies [20]. Other viral factors could also lead to ancestral or modern characteristics. Virus co-culture was positive in 6 out of 8 patients with a modern viral genotype (included in the LTNP-VC and -NC); whereas a viral isolate was recovered from only one of 8 patient with ancestral viral genotype (LTNP-EC and -VC) [20]. Proviral loads were significantly higher in LTNPs with modern versus patients with ancestral viral dating. Viral replication capacity as well as viral fitness may also contribute to the clinical presentation. Earlier work has shown that replication capacity of full primary viral isolates predict viral set-points after stopping treatment and also correlates with baseline *env* diversity [9], [27]. Recent work has shown the reduced replication capacity of chimeric viruses with *gag*-protease from LTNP-EC in comparison with chronically infected individuals [28]. Moreover envelope glycoproteins from virus from LTNP-EC exhibit a reduced entry capacity [29].

In conclusion, frequently used clinical definitions of patterns of disease progression are supported by the pattern of enrichment of validated host genetic markers and virological factors. This is particularly relevant for the standardization of definitions, in particular for rapid progression –a spectrum of disease that has been incompletely investigated, as well as for the discrimination within viremic controllers LTNPs. Host genetic and viral markers are also of use to the identification of incongruent assignments, i.e., individuals, with host or viral characteristics inconsistent with the clinical profile, who may signal novel pathogenic factors or mechanisms. This was indicated by Emu *et al*. [30] who underscored the importance of individuals with a LTNP status that lack any of the recognized protective factors. The study also emphasizes the need to apply a standardized and accepted set of clinical definitions for the purpose of disease stratification and research.

## Supporting Information

Table S1 Host genetic and viral results. For SNPs, 1 indicates the most common and 2 the variant allele. CCR5 haplotypes are presented according to published nomenclature, where CCR5 P1 is included in HHE, HHG*1 and HHF*1 (HHG*2 and HHF*2 were not considered as they include the protective alleles CCR5 Delta 32 and CCR2 V64I). Observed protective HLA alleles included B*2705, B*5701, B*5101, B*1302, A10 serogroup (A*2501, A*2601) and A*3201. Risk HLA alleles included B*35Px (B*3503), B22 serogroup (B*55, B*56), B*1801, A*2402 and A*2301. Participants are classified in the different classes according to the log of median viral load (Log VL), except for rapid progressors. For this class, individuals were ordered decrescendo according to time from seroconversion to CD4 <350. For elite controllers, the occasional blip was not considered for estimation of median Log VL. * non-B subtype viruses do not allow viral dating and X4/R5 genotyping. Score: simple additive genetic score that includes the most valuable genetic markers as explained in Materials and Methods. Quantification of DNA viral load, estimated by limiting dilution PCRs, was expressed as number of copies per million PBMCs. + charge: number of positive charged amino acids in the V3 loop. Ancest: Ancestral; Cauc: Caucasian; N.D.: not done; N.A.: not available. Mode HIV = mode of HIV acquisition; MSM: men having sex with men, HET: heterosexual, IDU: intravenous drug use. Green: protective factors; red: risk factors.
(1.20 MB PDF)
