# Supplementary material for: Host and Viral Genetic Correlates of Clinical Definitions of HIV-1 Disease Progression
Source: PLoS One. 2010 Jun 11;5(6):e11079. doi: 10.1371/journal.pone.0011079 (PMC2884031; doi:10.1371/journal.pone.0011079)
Supplement: Table S1 — Host genetic and viral results. For SNPs, 1 indicates the most common and 2 the variant allele. CCR5 haplotypes are presented according to published nomenclature, where CCR5 P1 is included in HHE, HHG*1 and HHF*1 (HHG*2 and HHF*2 were not considered as they include the protective alleles CCR5 Delta 32 and CCR2 V64I). Observed protective HLA alleles included B*2705, B*5701, B*5101, B*1302, A10 serogroup (A*2501, A*2601) and A*3201. Risk HLA alleles included B*35Px (B*3503), B22 serogroup (B*55, B*56), B*1801, A*2402 and A*2301. Participants are classified in the different classes according to the log of median viral load (Log VL), except for rapid progressors. For this class, individuals were ordered decrescendo according to time from seroconversion to CD4 <350. For elite controllers, the occasional blip was not considered for estimation of median Log VL. * non-B subtype viruses do not allow viral dating and X4/R5 genotyping. Score: simple additive genetic score that includes the most valuable genetic markers as explained in Materials and Methods. Quantification of DNA viral load, estimated by limiting dilution PCRs, was expressed as number of copies per million PBMCs. + charge: number of positive charged amino acids in the V3 loop. Ancest: Ancestral; Cauc: Caucasian; N.D.: not done; N.A.: not available. Mode HIV = mode of HIV acquisition; MSM: men having sex with men, HET: heterosexual, IDU: intravenous drug use. Green: protective factors; red: risk factors. (1.20 MB PDF) [file pone.0011079.s001.pdf]

Supplemetary Table 1

	Patient	CCR5 del32 rs333	CCR2 V64I rs1799864	CCR5 haplotype		CCR_P1 rs1799988	CCL3L1 (n)	HCP5 rs2395029	HLA-C rs9264942	ZNRD1 rs3869068	HLA-A		HLA-B		Score	Vial Dating	Log VL	Proviral	Pred pheno	11,25 V3 residues	+ charge	net charge	Race	Age	Gender	Mode HIV
LTNP-EC	1	11	11	HHC	HHE	P1	3	11	22	11	0201	0205	2705	5801	3	ANCEST	<1.7	1	R5	SE	7	5	Cauc	53	M	IDU
	2	11	11	HHC	HHE	P1	3	11	22	11	2402	6801	2705	4402	3	ANCEST	<1.7	1	R5	GE	7	5	Cauc	35	F	HET
	3	11	12	HHC	HHG*2		1	11	12	11	0201	2902	1404	4402	2	ANCEST	<1.7	2	R5	SE	6	5	Cauc	35	M	IDU
	4	12	11	HHC	HHG*2		3	11	12	11	0201	2902	3901	4402	2	ANCEST	<1.7	55	R5	SE	7	5	Cauc	42	F	HET
	5	11	12	HHE	HHF*2	P1	2	12	22	11	1101	3002	2705	5701	5	ANCEST	<1.7	1	R5	GE	6	4	Cauc	43	M	MSM
	6	12	12	HHG*2	HHF*2		2	11	12	11	0201	1101	4402	5601	2	ANCEST	<1.7	2	R5	SE	7	5	Cauc	46	M	IDU
	7	12	12	HHG*2	HHF*2		2	11	22	11	0201	6801	2705	3503	4	ANCEST	<1.7	4	R5	SE	6	4	Cauc	47	M	IDU
	8	11	11	HHC	HHE	P1	2	12	22	11	0101	0201	1402	5701	3	ANCEST	<1.7	2	R5	SK	8	7	Cauc	51	F	IDU
	9*	11	11	HHC	HHG*1	P1	1	12	22	12	0201	3101	3901	5701	3	N.D.	<1.7		N.D.	N.D.	N.D.	N.D.	Cauc	52	F	IDU
LTNP-VC	10	11	11	HHC	HHG*1	P1	3	11	12	11	0201	2401	1501	2705	2	ANCEST	2.03	3	R5	SE	7	5	Cauc	55	F	IDU
	11	11	11	HHC	HHE	P1	4	11	12	11	0201	1101	0702	5201	1	ANCEST	2.41	3	R5	SE	7	5	Cauc	47	M	IDU
	12	11	11	HHC	HHC		2	11	12	11	0101	1101	0801	2705	2	MODERN	2.53	4	R5	SD	6	4	Cauc	49	F	IDU
	13	11	11	HHC	HHG*1	P1	2	11	12	11	0201	0201	2705	4901	1	MODERN	2.65		R5	SR	5	4	Cauc	39	M	MSM
	14	12	11	HHE	HHG*2	P1	3	11	11	11	0103	0201	3924	4102	1	ANCEST	2.76	4	R5	GE	7	5	Cauc	40	M	IDU
	15	12	11	HHC	HHG*2		2	11	11	12	2601	2902	4403	4901	2	MODERN	2.87	16	R5	SE	7	6	Cauc	46	M	IDU
	16*	12	11	HHC	HHG*2		2	11	12	11	1101	1101	1401	5501	1	N.D.	3.07		N.D.	N.D.	N.D.	N.D.	Cauc	45	M	MSM
LTNP-NC	17	11	12	HHC	HHF*2		1	11	22	12	2501	3201	1801	5801	2	MODERN	3.46	30	R5	GD	8	6	Cauc	35	M	IDU
	18	12	11	HHC	HHG*2		2	11	12	11	0101	2902	1402	4403	2	MODERN	3.47	7	R5	SE	7	5	Cauc	48	M	IDU
	19	11	11	HHE	HHE	P1/P1	2	11	12	12	0201	2501	0702	5801	-1	MODERN	3.52		R5	SD	6	4	Cauc	31	F	IDU
	20	11	11	HHC	HHC		2	11	12	12	3002	3101	1501	1801	0	MODERN	3.62	98	R5	SE	8	6	Cauc	62	M	MSM
	21	11	11	HHC	HHC		3	11	22	11	0201	0201	2705	3801	3	MODERN	3.64	100	R5	SE	6	4	Cauc	62	M	MSM
	22	11	11	HHE	HHE	P1/P1	2	11	11	12	1101	2601	1501	3501	-2	MODERN	3.88	110	R5	SQ	6	5	Cauc	56	M	MSM
	23	12	12	HHG*2	HHF*2		3	11	12	11	0201	2301	4402	4901	3	MODERN	3.89	135	R5	SD	7	5	Cauc	43	M	IDU
	24	12	11	HHC	HHG*2		2	11	22	12	2601	2902	3801	4501	3	MODERN	3.98		R5	SQ	6	5	Cauc	42	F	IDU
	25	12	12	HHG*2	HHF*2		1	11	11	12	0201	2601	4403	5101	3	MODERN	4.15	34	R5	SG	6	5	Hispanic	44	M	MSM
	26	11	11	HHC	HHC		2	11	12	11	0301	2301	4002	4403	1	MODERN	4.2	194	R5	SA	6	5	Cauc	42	M	MSM
	27	11	11	HHE	HHE	P1/P1	2	11	12	11	1101	2902	0702	5207	-1	MODERN	4.29		R5	GE	7	5	Cauc	42	F	IDU
	28	12	12	HHG*2	HHF*2		2	11	11	11	0201	1101	0702	0702	2	MODERN	4.34		R5	SD	5	4	Cauc	41	F	IDU
	29	11	11	HHC	HHC		3	12	11	11	0101	0301	3501	5701	1	MODERN	4.56	89	R5	GE	6	4	Cauc	44	M	MSM
	30	11	11	HHC	HHE	P1	3	12	22	11	0201	0201	5101	5701	4	MODERN	4.62		R5	SQ	7	6	Cauc	50	M	MSM
P	31	11	12	HHE	HHF*2	P1	1	11	11	11	0201	0301	1801	3504	0	MODERN	4.23		R5	SD	7	5	Cauc	40	F	IDU
	32	11	11	HHC	HHE	P1	1	11	12	11	0301	1101	1401	5101	1	MODERN	4.45		R5	SE	6	4	Cauc	39	F	HET
	33	11	11	HHA	HHC		2	11	11	11	0201	0201	3503	4402	-1	MODERN	4.5		R5	SD	6	4	Cauc	43	M	MSM
	34	11	11	HHC	HHG*1	P1	3	12	12	12	0101	3303	3508	5701	2	MODERN	4.69						Cauc	52	F	IDU
	35	11	11	HHC	HHE	P1	1	11	12	11	3201	6801	0801	4501	1	MODERN	4.72		R5	SD	8	6	Cauc	50	M	MSM
	36	11	11	HHA	HHA		2	11	22	11	0201	0201	2702	2705	3	MODERN	4.84		R5	SD	7	5	Cauc	61	M	MSM
	37	11	11	HHC	HHC		1	11	11	11	0301	1101	3501	3503	-1	MODERN	4.94						Cauc	37	M	MSM
	38	11	11	HHC	HHC		1	11	11	12	0201	2601	0702	1501	0	MODERN	5.04		R5	SD	6	4	Cauc	51	M	MSM
	39	11	12	HHF*2	HHG*1	P1	2	11	11	11	0201	0201	3502	4001	1	MODERN	5.21		R5	SN	8	7	Cauc	33	M	IDU
	40	12	11	HHE	HHG*2	P1	2	11	22	11	2402	2402	3901	4405	3	MODERN	5.22		R5	SD	8	6	Cauc	42	M	MSM
RP	41	11	11	HHE	HHE	P1/P1	3	11	11	11	0201	0201	0702	4001	-2	MODERN	N.A.		R5	SD	6	4	Cauc	63	M	MSM
	42	11	11	HHC	HHE	P1	2	11	11	11	0301	0301	0702	1518	0	MODERN	5	225	R5	SE	7	5	Cauc	46	M	MSM
	43	11	11	HHC	HHC		3	11	11	11	2402	2902	0702	4403	0	MODERN	5.35	346	R5	SA	6	5	Cauc	45	M	MSM
	44	11	11	HHC	HHC		1	11	12	12	0201	2601	3801	1801	0	MODERN	N.A.						Cauc	45	M	MSM
	45	11	12	HHA	HHF*2		2	11	11	11	0101	6801	0801	3501	1	MODERN	5.5		R5	SD	6	4	Cauc	44	M	IDU
	46	11	11	HHA	HHC		2	11	11	11	0101	2402	0801	1801	-1	MODERN	5.87		R5	SD	7	5	Cauc	33	F	IDU
	47	11	11	HHC	HHG*1	P1	2	11	11	11	0201	0301	0702	1801	-1	MODERN	N.A.		R5	SQ	6	5	Cauc	62	M	MSM
	48	11	12	HHC	HHF*2		2	11	11	11	3201	6901	3501	3503	0	MODERN	N.A.						Cauc	56	M	HET
	49	11	11	HHC	HHC		2	11	11	11	0101	2902	0801	3501	0	MODERN	N.A.		R5	SD	7	5	n	43	M	MSM
	50	11	11	HHC	HHE	P1	3	11	12	11	0101	0201	0702	1302	2	MODERN	N.A.		R5	GQ	8	7	Cauc	43	M	HET
	51	11	11	HHA	HHE	P1	3	11	11	11	0201	0301	0702	0702	0	MODERN	N.A.		R5	GQ	6	5	Cauc	36	M	MSM
	52	11	12	HHC	HHF*2		2	11	11	12	0201	2901	0705	4001	1	MODERN	N.A.		R5	GE	6	4	Cauc	42	M	IDU
	53	11	11	HHC	HHG*1	P1	3	11	11	11	0201	2402	1501	5501	-1	MODERN	4.62						Cauc	62	M	MSM
	54	11	11	HHC	HHE	P1	3	11	11	11	0201	0301	0702	0702	0	MODERN	5.09						Cauc	67	M	HET
	55	11	12	HHC	HHF*2		2	11	12	12	0201	2601	1801	4403	0	MODERN	N.A.		R5	SE	6	4	Cauc	38	M	MSM
	56	11	11	HHC	HHE	P1	2	12	12	12	0201	6601	4102	7301	1	MODERN	N.A.		R5	SD	7	5	Cauc	39	M	HET
	57	11	11	HHE	HHG*1	P1/P1	3	11	11	11	0101	0301	0801	0801	-2	MODERN	4.5						Cauc	59	M	HET
	58	12	11	HHC	HHG*2		2	11	11	11	2402	6801	1501	4402	1	MODERN	N.A.		R5	SE	5	3	Cauc	45	M	HET
	59	11	11	HHC	HHC		2	11	11	11	2402	2902	1801	4403	-1	MODERN	N.A.		R5	GG	7	6	Cauc	52	M	MSM
	60	11	11	HHC	HHE	P1	2	11	12	11	0201	1101	1302	5301	1	MODERN	>5.70	295	R5	GE	6	4	Cauc	28	M	MSM
	61	11	11	HHC	HHE	P1	1	11	11	12	0201	3001	1801	3501	-1	MODERN	6.18						Cauc	34	F	IDU
	62	11	11	HHC	HHC		3	11	11	11	0101	6801	0801	3503	-1	MODERN	>5.70	450	R5	SE	6	4	Cauc	48	M	MSM+IDU
	63	11	11	HHC	HHE	P1	1	11	12	11	0101	1101	3701	5501	0	MODERN	4.96		R5	SA	6	5	Cauc	45	M	MSM
	64	11	11	HHE	HHE	P1/P1	2	11	11	11	0101	0202	0801	5301	-3	MODERN	5.02	40	R5	SE	6	4	Cauc	35	M	MSM
